# Early cardiac morphogenesis defects caused by loss of embryonic macrophage function in *Xenopus*

**DOI:** 10.1016/j.mod.2011.04.002

**Published:** 2011-05

**Authors:** Stuart J. Smith, Timothy J. Mohun

**Affiliations:** Division of Developmental Biology, MRC-National Institute for Medical Research, The Ridgeway, Mill Hill, London NW7 1AA, UK

**Keywords:** *Xenopus* heart development, Myeloid, Macrophage, Spib, Myocardium, Morphogenesis

## Abstract

The heart-forming mesoderm in *Xenopus* embryos lies adjacent to the source of the first embryonic population of macrophages. Such macrophages underlie the bilateral myocardial cell layers as they converge to form a linear heart tube. We have examined whether such macrophages participate in early cardiac morphogenesis, combining morpholino oligonucleotides that inhibit macrophage differentiation or function with transgenic reporters to assess macrophage numbers in living embryos. We show that loss of macrophage production through tadpole stages of development by morpholino-mediated knockdown of the *spib* transcription factor results in an arrest of heart formation. The myocardium fails to form the fused, wedge-shaped trough that precedes heart tube formation and in the most severe cases, myocardial differentiation is also impaired. Knockdown of the Ly6 protein *lurp1*, an early, secreted product from differentiated macrophages, produces a similar arrest to myocardial morphogenesis. Heart development can moreover be rescued by surgical-transfer of normal macrophage domains into morpholino-injected embryos. Together, these results demonstrate that amphibian heart formation depends on the presence and activity of the macrophage population, indicating that these cells may be an important source of growth cues necessary for early cardiac morphogenesis.

## 1 Introduction

A universal and early step in vertebrate cardiogenesis is the formation of a linear heart tube, from which the mature chambered organ is derived. Whilst considerable progress has been made in understanding subsequent steps in chamber formation and the contribution of cells that initially lie outside the heart tube (Boogerd et al., 2009; Buckingham et al., 2005), much less is known about the morphogenetic events that regulate tube formation itself.

The contractile, myocardial cells of the heart tube are derived from bilateral, anterior lateral plate (splanchnic) mesodermal domains that converge on the ventral midline. In amphibian embryos, such domains have been termed the heart field, referring to classical embryological studies demonstrating the regulative capacity of such tissue in its contribution to the embryonic heart (reviewed in Mohun et al., 2003). In *Xenopus laevis*, myocardial precursors within the heart field express markers of striated muscle differentiation (stage 27) prior to fusion of the bilateral domains, some 9 h before formation of the heart tube (stage 32) (Latinkic et al., 2004; Mohun et al., 2000). Furthermore, as the bilateral precursors approach the ventral midline, they overlap with a second and distinct population of cells, the embryonic macrophages. This myeloid domain is also mesodermal in origin and initially encompasses the position on the ventral midline where the heart will ultimately form (Smith et al., 2002). Whilst macrophages begin to disperse from this site of origin prior to differentiation of cardiac precursors, they remain prevalent in the heart-forming region, filling the trough-shape made as the myocardium begins to fold into a tube (stage 29) and only become less numerous within the heart as a distinct endocardial population becomes evident (stages 30–31).

Such a first wave of migratory, “primitive” macrophages is also universal in development, providing an innate immune function to embryos. In addition to this role, there is also evidence that macrophages may play an important role in tissue and organ morphogenesis, although the precise nature of their contribution is poorly understood (reviewed in Ovchinnikov, 2008). For example, the osteopetrotic (*op*) mouse that lacks the principal growth factor for macrophages, *Csf1*, displays numerous organ defects that can be attributed to a lack of macrophage participation within developing tissues (Banaei-Bouchareb et al., 2004; Van Nguyen and Pollard, 2002). Macrophages are certainly responsible for phagocytic clearance of apoptotic corpses during embryogenesis (Henson and Hume, 2006) and one suggestion is that they can provide growth support, or a “trophic role” during formation of complex organ structures (Pollard, 2009). Consistent with this, transcriptional profiling indicates that macrophages synthesize a wide array of secreted proteins, including cytokines, growth factors and VEGFs (Rae et al., 2007), which could be deposited in discrete embryonic locations. The clearest picture of migratory macrophage involvement in early embryogenesis has been described in *Drosophila* larvae that possess equivalent cells called plasmatocytes (a class of hemocyte). In *pvr* mutant larvae that lack the sole VEGF/PDGF receptor in flies, plasmatocyte migration and viability are critically impaired (Bruckner et al., 2004). The lack of macrophage function is lethal, with a substantial loss of macrophage ECM deposition causing widespread morphogenesis defects including abnormalities to the ventral CNS (Olofsson and Page, 2005).

The overlapping location of myeloid and cardiac domains immediately prior to and during heart tube formation in *Xenopus* embryos raises the possibility that macrophages may play some role in early cardiac morphogenesis. To test this possibility, we have used morpholino oligonucleotide-mediated gene knockdown to interfere with macrophage differentiation or function and examined the effect on heart formation. Our results demonstrate that amphibian heart morphogenesis is indeed critically dependent upon the presence and activity of the primitive macrophage population.

## 2 Results

### 2.1 Spib-mediated macrophage production and embryonic heart formation appear linked

**Supplementary Fig. 1:**
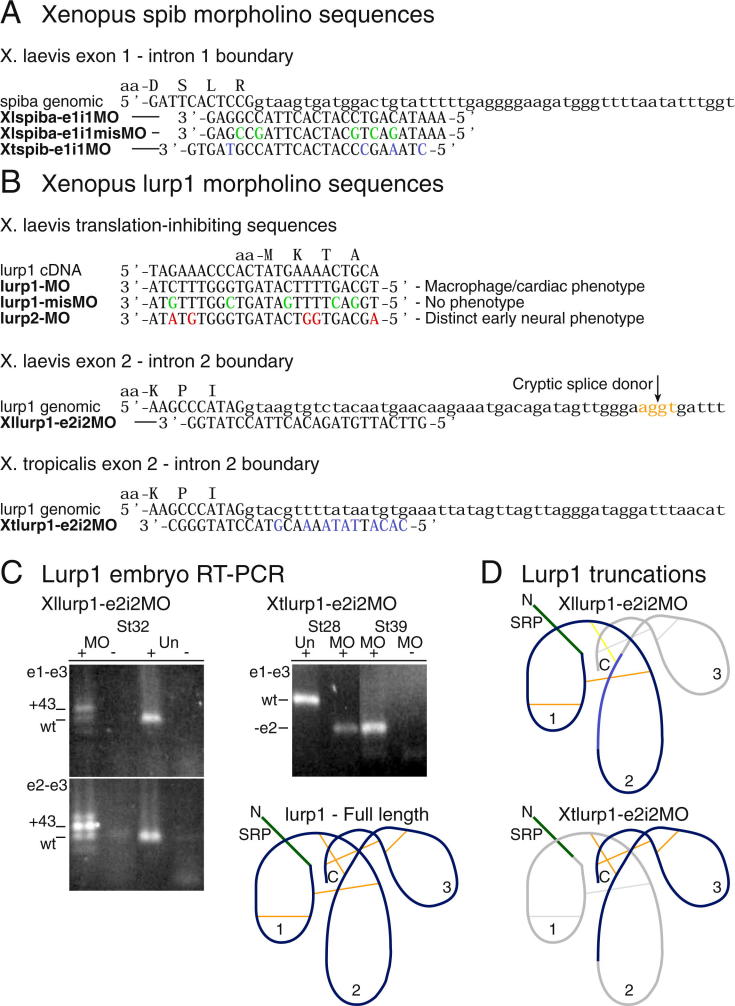
*Morpholino sequences and targets*. (A) Sequence of the *X. laevisspiba* gene exon 1-intron 1 splice boundary, with exon sequence uppercase and intron sequence lowercase. The morpholino Xlspiba-e1i1MO was designed against this boundary while the Xtspib-e1i1MO (Costa et al., 2008) matches the equivalent *X. tropicalis* boundary and contains four mismatches (blue text) with *X. laevisspiba*. The Xlspiba-e1i1misMO contains five distributed mismatches (green text). (B) Translation-inhibiting and splice-interfering morpholinos were designed to the *lurp1* gene. The lurp1-MO targets the start codon of *X. laevislurp1* mRNA, while the lurp1-misMO contains five evenly distributed mismatches (green text). The lurp2-MO was designed to a related Ly-6 sequence (Image: 6872870), containing five different mismatches (red text). The splice-interfering morpholino Xllurp1-e2i2MO designed against the exon 2-intron 2 boundary of *X. laevislurp1* forces utilization of an alternative splice donor site (orange text). Consequently, 43 bases of intron sequence are included within the *lurp1* mRNA and encode a premature stop codon. In *X. tropicalislurp1*, the exon 2-intron 2 boundary region does not contain a cryptic splice donor site and hence, the morpholino Xtlurp1-e2i2MO induces deletion of the 120 base exon 2 from the mRNA. There are ten mismatches between Xtlurp1-e2i2MO (blue text) and the *X. laevis* splice boundary. **C:** RT-PCR of *lurp1* from a single stage 32 *X. laevis* embryo that received 32 ng Xllurp1-e2i2MO injected into dorsal blastomeres (MO), plus uninjected control (Un). The e1-e3 PCR product used oligonucleotide primers that reside in exons 1 and 3 (L1-1735/L1-1740) while the e2-e3 PCR product used primers from exons 2 and 3 (L1-1737/L1-1740). *Lurp1* RT-PCR from *X. tropicalis* embryos injected with 8 ng Xtlurp1-e2i2MO into 2/2 blastomeres (MO), plus an uninjected control (Un). Exon 2-deletion is detected at stage 28 and also stage 39 (primers L1-1469/L1-1470). +/-, denotes addition of reverse transcriptase. PCR products were sequenced to confirm their identity. **D:** Truncated lurp1 peptides produced by the splice-interfering morpholinos are depicted superimposed onto a cartoon of a three-finger protein structure. The Xllurp1-e2i2MO-induced peptide contains finger 1 and half of finger 2 only (dark blue), then five amino acids encoded by intron 2 (pale blue). The deleted sequence is represented grey. The Xtlurp1-e2i2MO-induced peptide is missing the normal cleavage site of the signal recognition peptide (SRP, green) and also fingers 1 and 2. The five cysteine bridges of full length lurp1 are represented (orange).

The ets transcription factor, *spib*, is essential for differentiation of the primitive macrophage population in *Xenopus* embryos (Costa et al., 2008). We therefore used morpholino oligonucleotides directed against *spib* to test whether inhibition of macrophage differentiation affected embryonic heart formation in *X. laevis*. Two RNA splice-interfering morpholinos were used in our studies: The Xtspib-e1i1MO, originally designed to target the *X. tropicalis* gene (Costa et al., 2008) and Xlspiba-e1i1MO matches the *X. laevis* *spiba* allele sequence (Suppl. Fig. 1). Each proved similarly effective in inhibiting the initial differentiation and dispersal of macrophages during tailbud stages. In both cases, a proportion of later stage tadpoles recover macrophage number, presumably due to progressive reduction in effective morpholino concentration.

**Supplementary Fig. 2:**
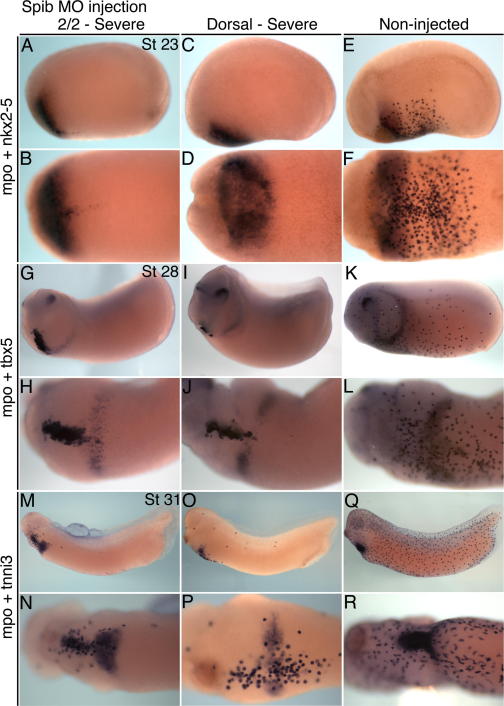
*Spib morpholino comparison of bilateral versus dorsal blastomere injection.* (A–F): Tailbud stage 23 embryos injected with Xtspib-e1i1MO, 40 ng dose, into both blastomeres at the two-cell stage (2/2, A and B), into dorsal blastomeres (C and D), or non-injected control (E and F). Embryos hybridized with probes for *mpo* and *nkx2-5*. (G–L): Late tailbud stage 28 embryos, with the same sequence of *spib* morpholino blastomere injections presented and hybridized to *mpo* and *tbx5*. (M–R): Stage 31 tadpoles with the same sequence of *spib* morpholino blastomere injections and hybridized to *mpo* and *tnni3*. Different morpholino injected embryos were chosen for presentation compared to Fig. 2 and Fig. 3.

Morpholinos were injected into *X. laevis* embryos that carried the *Tg[lurp1:egfp]* transgene (Smith et al., 2002, 2007) to enable production of early macrophages to be monitored in living embryos. This transgene is active throughout development in all myeloid cells, including all the primitive macrophages and yields visible eGFP fluorescence by stage 22 as they begin migration (reporter mRNA can be detected earlier at stage 18, plus there is also limited neural expression). Using *Tg[lurp1:egfp]* embryos allowed the effectiveness of morpholino treatment to be correlated with subsequent phenotype. Injection into dorsal blastomeres ensured that morpholinos were localized to a region of the embryo that included the myeloid mesoderm and the heart. Parallel injections of the ventral blastomeres were used as controls for assessing phenotypes. Injections into both blastomeres at the two-cell stage were also performed and gave identical phenotypes, albeit at a slightly reduced incidence (Suppl. Fig. 2, see Experimental Procedures).

**Fig. 1:**
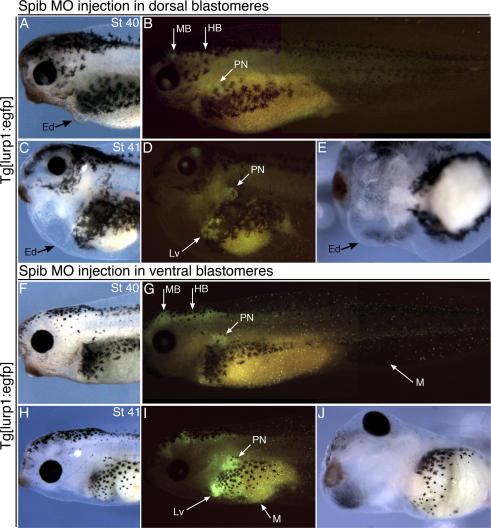
Heart defect in a tadpole after sustained spib morpholino inhibition of myeloid cell differentiation. (A–E) *X. laevisTg[lurp1:egfp]* tadpole that was injected with Xtspib-e1i1MO into dorsal blastomeres, 40 ng dose. Tadpole was photographed at stage 40 (A and B) and stage 41 (C–E), with fluorescence from eGFP shown (B and D). The complete inhibition of myeloid cell differentiation caused by loss of *spib* has been sustained to stage 40. A large edema formed during this time interval, indicative of cardiac dysfunction (A, C, E). (F–J) Control sibling tadpole that was injected with the same morpholino into ventral blastomeres, photographed at stage 40 (F and G) and stage 41 (H–J). Fluorescent embryonic macrophages can be seen distributed throughout the tadpole (G and I). Hematopoiesis also observed in pronephros and liver (G and I). No edema developed and heart morphology appeared normal. Anterior is to the left in lateral (A–D, F–I) and ventral (E and J) views. Ed, edema; M, myeloid/macrophage; PN, pronephros; Lv, liver; MB and HB, additional domains of *Tg[lurp1:egfp]* fluorescence in mid and hindbrain.

Initial experiments showed that a high proportion of embryos receiving morpholino into both dorsal blastomeres developed with serious heart malformations (67% of total), frequently resulting in tadpoles without an obvious heart at all (38%) (Suppl. Table 1). Using the transgenic marker to visualise macrophages, it was evident that sustained loss of macrophage production through to tadpole stages correlated with severe heart malformation (100%), yielding a spectrum of phenotypes including complete loss of a discernible heart (Fig. 1, Suppl. Table 1). Whilst outwardly largely normal at stage 40 (Fig. 1A and B), such tadpoles subsequently rapidly developed edema typical of cardiovascular dysfunction (Fig. 1C–E). In contrast, those embryos showing only a temporary reduction of macrophage numbers during tailbud stages subsequently showed more normal cardiogenesis (Suppl. Table 1). As a control, the same morpholino injected into (both) ventral blastomeres never affected macrophage production and the tadpoles formed with beating hearts (Fig. 1F–J).

**Supplementary Fig. 3:**
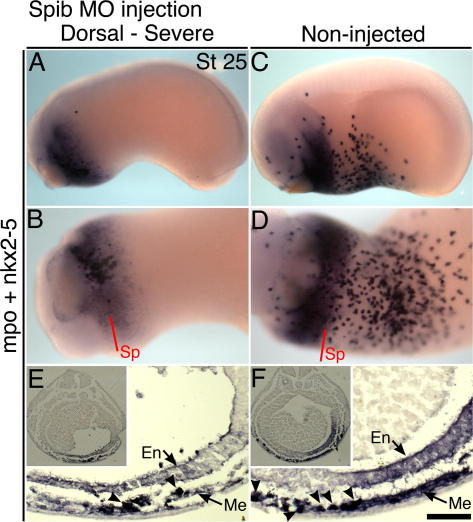
*Mesodermal nkx2-5 expression is detected in spib morpholino embryos*. (A–D): Stage 25 embryo injected with the Xtspib-e1i1MO into dorsal blastomeres, 40 ng dose (A and B), or non-injected control (C and D). Embryos hybridized to *mpo* and *nkx2-5*. (E and F) Transverse sections through the heart field of the embryos. Inset image shows the whole section while main image depicts the region of the ventral midline. Arrowheads indicate dark *mpo* stain (E and F). NFR-counterstained. Scale bar = 100 μm. Sp, section plane; Me, mesoderm; En, endoderm.

**Fig. 2:**
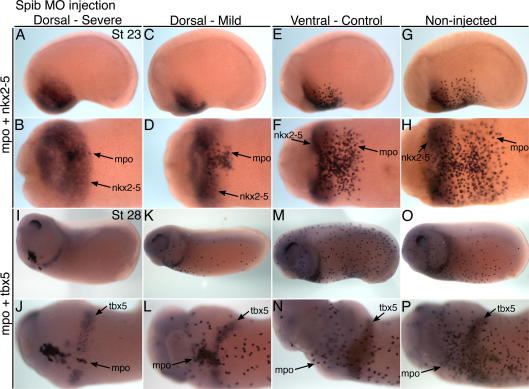
Spib morpholino affects myeloid cell production but not cardiac specification. (A–D) Tailbud stage 23 embryos injected with the Xtspib-e1i1MO into dorsal blastomeres (A–D), 40 ng dose, or as a control in ventral blastomeres (E and F), or non-injected sibling (G and H). Embryos subjected to wholemount *in situ* hybridization for *mpo* (myeloid/macrophage) and *nkx2-5* (heart field). Dorsal-injected embryos exhibited either a severe loss of the myeloid domain (A and B) or a milder reduction (C and D). *Nkx2-5* mRNA expression detected in all examples. (I–P) Late tailbud stage 28 embryos injected with the *spib* morpholino, 40 ng dose and with the same sequence of blastomere injections presented. Embryos hybridized with probes for *mpo* and *tbx5*. Cardiac *tbx5* mRNA detected in all examples. Anterior is to left, lateral views and ventral view of heart fields.

In order to investigate the nature of the link between cardiogenesis and macrophage production, we assayed embryos fixed at tailbud stages of development. Wholemount *in situ* hybridization for *mpo* (*pox2*) expression was used, since it provides an early and robust marker of primitive macrophage differentiation from the anterior myeloid domain in *Xenopus* (normally detectable from stage 18). Some variability of the *spib* morpholino effects was noted at these stages. At stage 22, a severe dorsal blastomere phenotype occurred where the near complete loss of *mpo* expression was observed (30% of embryos), indicating profound inhibition of embryonic myeloid differentiation (Fig. 2A and B). More commonly, limited myeloid cell differentiation proceeded from a domain that was reduced in size (70% of embryos) (Fig. 2C and D) and dispersal of the macrophages was less extensive when compared with control embryos (Fig. 2E–H, Suppl. Table 2). With both severe and mild macrophage phenotypes, expression of the early cardiac transcription factor, *nkx2-5* was readily detected, indicating normal specification of the heart field in the ventro-lateral mesoderm adjacent to the macrophage population (Fig. 2A–D), albeit with some broadening of the cardiac domain. Histological analysis performed on stage 25 embryos confirmed that *nkx2-5* expression was retained in both cardiac mesoderm and underlying pharyngeal endoderm cell layers (Suppl. Fig. 3). In late tailbud stage embryos, the division into severe and milder macrophage phenotypes was generally retained (Fig. 2I–L). At stage 28, the heart-forming mesoderm additionally expresses *tbx5* in a pattern that extends in a dorsal and posterior direction towards progenitors of the future cardiac inflow regions (the sinus venosus and common cardinal veins). Moreover, this induction of cardiac *tbx5* expression proceeded as normal, despite the inhibition of myeloid cell differentiation (Fig. 2I–P).

**Fig. 3:**
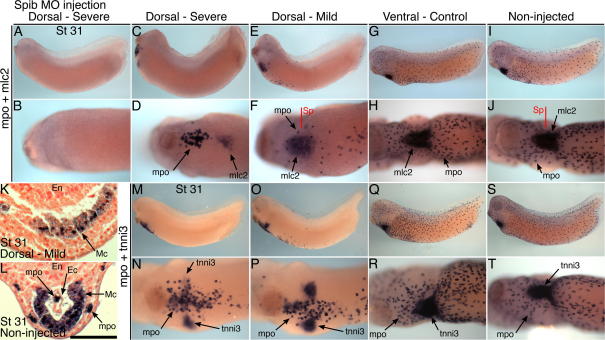
Spib morpholino affects myeloid cell production and also cardiac muscle morphogenesis. (A–J) Stage 31 tadpoles injected with Xtspib-e1i1MO into dorsal blastomeres (A–F), 40 ng dose, or control ventral blastomeres (G and H), or non-injected sibling (I and J), hybridized with probes for *mpo* and *mlc2* (myocardium). Two tadpoles with severe macrophage deficit are shown (A–D) and one with a milder reduction (E and F). Myocardial phenotype severity appears linked to the number of functional macrophages produced. Anterior is to left in lateral views and ventral view of heart-forming regions. (K and L) Transverse section through the myocardium of the mild phenotype tadpole (E, F, K) compared with non-injected control (I, J, L), Nuclear-Fast Red counterstained (NFR). Section plane indicated (Sp). Scale bar = 100 μm. En, endoderm; Mc, myocardium; Ec, endocardium. (M–T) Stage 31 tadpoles injected with the *spib* morpholino, 40 ng dose and with same sequence of blastomere injections presented. Tadpoles hybridized to *mpo* and *tnni3* (myocardium). One tadpole is shown with severe macrophage deficit (M and N) and one with a milder reduction (O and P).

Beyond stage 30, many embryos escaped the myeloid differentiation block (Costa et al., 2008) caused by the *spib* morpholino (Suppl. Table 2) and tadpoles could be classified into severe, mild and normal groups (16%, 35% and 49%, respectively), based on the extent of myeloid cell differentiation and migration. Assay of stage 31 tadpoles showed an intriguing link between macrophage production and heart myocardial gene expression. Severe dorsal blastomere phenotypes commonly contained small patches of mpo-expressing cells retained within the myeloid domain and showed only very weak myocardial differentiation, as revealed by *in situ* hybridization for the myocardial muscle markers, *mlc2* and *tnni3* (3. C, D, M, N). This myocardium was positioned on the ventral midline or displaced bilaterally (bifida), depending on the posterior distance the affected myeloid domain extended into the heart field (Fig. 3C, D, M and N). Occasionally tadpoles were completely devoid of macrophages and also showed no myocardial differentiation (Fig. 3A and B).

The mild phenotype group contained reduced numbers of macrophages that had nevertheless successfully migrated to head and trunk locations and were associated with only modest reductions to heart myocardial differentiation (Fig. 3E, F, O and P). Transverse sections through these mildly affected embryos did, nonetheless, reveal defective folding of the myocardium prior to heart tube formation, with few endocardial cells also apparent (Fig. 3K and L). In contrast, stage 31 tadpoles that had received *spib* morpholino into ventral blastomeres had normal numbers of dispersed macrophages and formed a normal, triangular-shaped myocardial trough from the bilateral cardiac precursors (100% were normal) (Fig. 3G, H, Q and R). These results demonstrate that sustained loss of embryonic macrophages resulting from morpholino-mediated *spib* knockdown has a profound effect on formation of the heart in *Xenopus* embryos.

### 2.2 Spib is expressed in the myeloid but not in the myocardial lineage

**Fig. 4:**
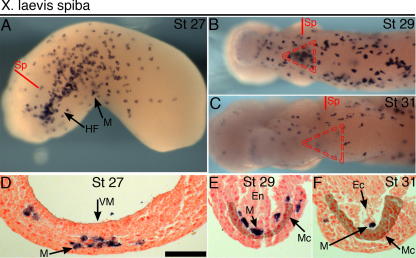
Spiba mRNA expression within the heart-forming region. (A–C) Wholemount *in situ* hybridization for myeloid *spiba*, ventral views of heart-forming region, at stage 27 (A), stage 29 (B) and stage 31 (C). Red triangle depicts position of forming myocardium (B and C). (D–F) Transverse sections through heart region of same embryos, with section planes depicted (A–C). Stage 27 (D), stage 29 (E) and stage 31 sections (F). To ease identification, the approximate position of the (myocardial) mesoderm has been artificially-darkened (E and F). NFR (D–F) nuclear counterstained. Scale bar = 100 μm. Sp, section plane; M, myeloid/macrophage; HF, heart field; Mc, myocardium; Ec, endocardium; VM, ventral midline; En, endoderm.

One possible explanation for the relationship between macrophage and myocardial phenotypes observed is that *spib* might participate directly within the myocardial lineage during *Xenopus* development. Since macrophage cells are intimately associated with the heart primordium, it is impossible to assay *spib* expression in RNA extracted from early myocardial tissue (e.g. RT-PCR). Our analysis by wholemount RNA *in situ* hybridization confirms previous findings (Costa et al., 2008) that no detectable *spib* gene expression was evident in the heart myocardium or its progenitors (Fig. 4A–F). Instead, *spib* expression marks the myeloid domain of the anterior ventral blood island (aVBI) and subsequently the migrating population of embryonic macrophages. *Spib*-expressing macrophages are typically found closely associated with the heart-forming region up to stage 29 (Fig. 4B and E), underlying the myocardium. Beyond this point, the cells are less numerous within the folding heart tube (Fig. 4C and F). The distinct pattern of myeloid cell distribution around the heart has been observed previously with other markers, *mpo* (*pox2*) and *lurp1* (Smith et al., 2002).

Such data cannot exclude the formal possibility that a low level of *spib* expression within myocardial cells or their precursors remains undetectable by *in situ* methods. As an alternative therefore, we sought a second means to disrupt the early macrophage population, in a way that could not target myocardial cells. To achieve this, we disrupted macrophage function rather than differentiation.

### 2.3 Lurp1-mediated macrophage function affects early cardiac morphogenesis

The small, secreted Ly6 protein, lurp1 is one of the best-characterized markers of the primitive macrophage lineage in *Xenopus*. Not only is expression restricted exclusively to embryonic myeloid cells, including solely the early macrophages at tailbud stages; similar specificity is obtained with the *lurp1* gene promoter in transgenic reporter experiments (Smith et al., 2002, 2007). Ly6 proteins share little sequence similarity among the diverse family members, but are nevertheless characterised by a conserved, three-finger core structure (Galat, 2008). Whilst few Ly6 proteins have been studied in detail, there is gathering evidence they can play essential extra-cellular roles during embryonic development (Hijazi et al., 2009). Since *lurp1* expression commences in newly differentiated embryonic macrophages, we hypothesized that the secreted protein is important in mediating macrophage functions. We therefore designed morpholinos to target *X. laevis* *lurp1*, either by inhibiting translation or interfering with RNA splicing (Suppl. Fig. 1).

**Supplementary Fig. 4:**
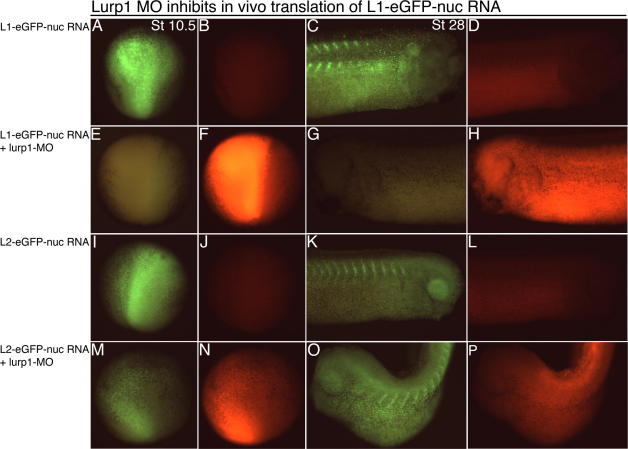
*Target specificity of lurp1 translation-inhibiting morpholino*. (A–D): Injection of 200 pg L1-eGFP-nuc RNA into one blastomere at the two-cell stage (1/2). Fluorescence viewed of same embryo at stage 10.5 (A and B) and stage 28 (C and D). (E–H): Similar injection of L1-eGFP-nuc RNA, followed by injection of 16 ng lurp1-MO (plus rhodamine-Dextran) to the same site at the four-cell stage. The lurp1-MO inhibits translation of the synthetic RNA *in vivo*. (I–L) Injection of 200 pg of control L2-eGFP-nuc RNA. (M–P) Similar injection of L2-eGFP-nuc RNA, followed by injection of 16 ng lurp1-MO. The lurp1-MO does not inhibit translation of the control RNA. Images of green fluorescence from eGFP and red fluorescence from rhodamine-Dextran are presented. Stage 10.5 embryos oriented with the blastopore at the bottom of each image while stage 28 embryos are lateral views.

The target specificity and effectiveness of the translation-inhibiting *lurp1* morpholino (lurp1-MO) was established *in vivo* using synthetic RNA that contained the 5′-most *lurp1* nucleotide sequences fused to eGFP. Lurp1-MO inhibited the translation of this injected synthetic RNA (Suppl. Fig. 4A–H) but had no effect on an equivalent RNA derived from the closest *Xenopus* Ly6 homologue, *lurp2*, which included five mismatched nucleotides (Suppl. Fig. 4I–P).

**Supplementary Fig. 5:**
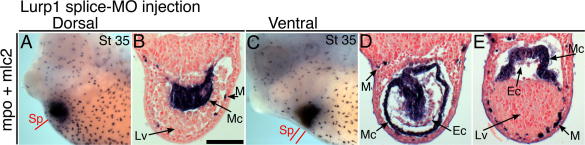
*Lurp1 RNA splice-interfering morpholino similarly affects myocardial morphogenesis*. (A and B) Stage 35 tadpole injected with Xllurp1-e2i2MO into dorsal blastomeres, 16 ng dose, hybridized to *mpo* and *mlc2*. The section plane (B) is indicated on the head (A). (C–E) Control tadpole with same morpholino in ventral blastomeres. Position of two sections (D andindicated (C). Scale bar = 100 μm. Sp, section plane; Mc, myocardium; Ec, endocardium; M, macrophage; Lv, liver.

**Fig. 5:**
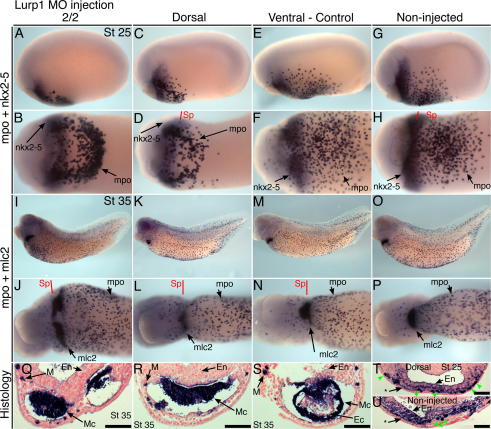
Lurp1 morpholino affects myeloid domain migration and cardiac muscle morphogenesis. (A–H) A stage 25 embryo injected with the lurp1-MO into both blastomeres at the two-cell stage (2/2) (A and B), 12 ng dose. Embryos similarly injected into dorsal blastomeres (C and D), or as a control in ventral blastomeres (E and F), or non-injected sibling (G and H) and hybridized with probes for *mpo* and *nkx2-5*. (I–P) Stage 35 tadpoles injected with the *lurp1* morpholino, 12 ng dose, with the same sequence of blastomere injections presented and hybridized to *mpo* and *mlc2*. Anterior is to the left in lateral and detail ventral views. (Q–S) Transverse section through the heart region of tadpoles injected with *lurp1* morpholino. Examples of a cardiac bifida phenotype (I, J, Q) and abnormal myocardial folding morphogenesis (K, L, R) were observed in both the two-cell stage and dorsal blastomere injection experiments but never after ventral blastomere injection (M, N, S). (T and U) Stage 25 heart field sections of embryo with dorsal blastomere morpholino (C, D, T) and non-injected sibling (G, H, U). Green arrowheads show dark *mpo* stain of macrophages. Asterisk (∗) denotes mesodermal *nkx2-5* stain. NFR-counterstained. Scale bar = 100 μm. Sp, section plane; M, myeloid/macrophage; En, endoderm; Mc, myocardium; Ec, endocardium.

Injection of the lurp1-MO into dorsal blastomeres (or 2/2-cell injection; Suppl. Table 3) had no effect on the timing of myeloid differentiation, which occurred normally in tailbud embryos; nor did it affect onset of *nkx2*-*5* expression in the cardiac mesoderm. However, the first differences with control embryos were detected at stage 25, with migration of macrophages being moderately delayed and occurring from more lateral positions than those of embryos injected in ventral blastomeres (Fig. 5A–H, T and U). Nonetheless by stage 35, dorsal blastomere injected tadpoles had fully dispersed macrophage populations indistinguishable from control embryos, but consistently showed a severe cardiac phenotype (100%). This ranged from apparent cardiac bifida (Fig. 5I and J) to profoundly abnormal midline-morphogenesis (Fig. 5K and L). Histological analysis revealed the extent of such malformations, demonstrating that the myocardial tube failed to form (Fig. 5Q and R). In contrast, control injection of the lurp1-MO into ventral blastomeres had no effect on the early stages of heart formation (Fig. 5M, N and S). Morpholinos that cause aberrant RNA splicing of *lurp1* yielded identical effects on myocardial morphogenesis, confirming specificity of the *lurp1* knockdown phenotype (Suppl. Figs. 1 and 5 and Table 3).

**Fig. 6:**
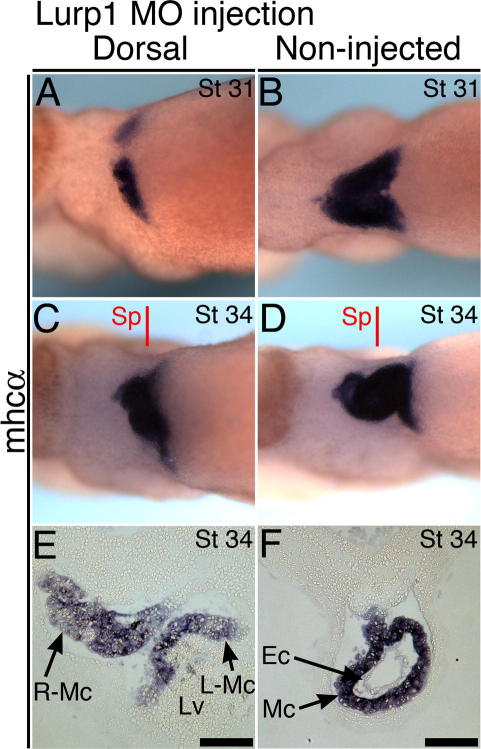
Lurp1 morpholino disrupts myocardial morphogenesis prior to heart tube formation. (A–D) Tadpoles injected with the lurp1-MO into dorsal blastomeres, 12 ng dose, at stage 31 (A) and stage 34 (C), compared with non-injected sibling embryos (B and D). Tadpoles hybridized to *mhcα* (myocardium). Anterior is to left in ventral view of heart-forming regions. (E and F) Heart section of the stage 34 tadpole with dorsal blastomere morpholino and flat, bilateral myocardium (C and E), compared with control stage 34 tadpole (D and F). No counterstain. Scale bars = 100 μm. Sp, section plane; L, left; R, right; Mc, myocardium; Ec, endocardium; Lv, liver.

Normal development of the trough-shaped myocardium that precedes the heart tube involves a combination of three processes; fusion of bilateral fields, elongation along the anterior–posterior axis and ventral-ward movement that physically separates the myocardium from the adjacent endoderm. These events occur between stages 29 and 31 (Fig. 4E and F, Fig. 6B), while macrophages are abundant within the heart-forming region. With the lurp1-MO however, myocardial morphogenesis arrest frequently involves failure of all three processes. At stage 31, the myocardium remains as narrow bilateral strips (Fig. 6, compare panels A and B). In stage 34–35 tadpoles, the myocardial cells can be detected on the ventral midline but proper anterior-posterior elongation does not occur (Fig. 5K and L, Fig. 6C). Even when fusion appears to succeed, histology often shows that left and right myocardial components remain separate (Fig. 6C and E). Moreover, the curved-shaped myocardium always remains in contact with the endoderm (Fig. 5Q and R, Fig. 6E). With no heart tube formed, the malformed myocardium subsequently becomes buckled at the posterior pole, presumably as a result of growth by the liver anlagen (Fig. 6E).

### 2.4 Endocardial smad3 expression is detected in macrophage-morpholino defective embryos

**Fig. 7:**
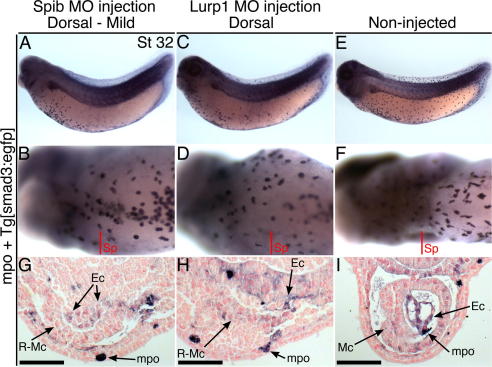
Endocardial smad3 expression is detected in macrophage-morpholino defective embryos. (A–F) Tadpoles injected into dorsal blastomeres with *spib* morpholino, 40 ng dose (A and B), or *lurp1* morpholino, 12 ng dose (C and D), or non-injected sibling (E and F). The *spib* morpholino caused a mild macrophage deficit phenotype (A and B) while the *lurp1* morpholino induced abnormal, broadened morphology at the ventral midline (D). Tadpoles carry the *Tg[smad3-egfp]* reporter and were hybridized with probes for *mpo* and eGFP. At stage 32, the transgenic line gives eGFP expression within head, eye, pronephros, somite and notochord domains (A, C, E), while strong expression occurs in the forming endocardium, but not in myocardium at these stages, nor in macrophages. Anterior is to left in lateral views and ventral view of heart-forming regions. (G–I) Transverse heart sections of the tadpoles. Cells underlying the malformed myocardium gave endocardium-type *smad3* reporter expression in morpholino tadpoles (G and H) while the forming endocardium stained strongly positive in the control (I). The right-sided myocardial region only, was presented for the morpholino injected tadpoles due to their broader ventral surface and to allow the necessary image magnification. NFR-counterstained. Scale bars = 100 μm. Sp, section plane; R, right; Mc, myocardium; Ec, endocardial cell activity.

Cells of the endocardium appear in the *Xenopus* heart-forming region from stage 30. Histological identification often relies on their rapid organization to form a lumen, central to the myocardial trough. Nonetheless, during this period, endocardial cells express significant levels of the TGF-β signalling molecule *smad3* (Howell et al., 2001). We made a transgenic *Tg[smad3:egfp]* reporter line to amplify the *smad3* signal and have analysed these embryos for the presence of endocardial gene expression after injection of *spib* and *lurp1* morpholinos (see Section 4). Our earlier analyses failed to identify a morphologically discrete endocardium structure in any embryos of the mildly affected *spib* morpholino phenotype group, nor when using the *lurp1* morpholino. However, in both cases, a diffuse region of *smad3* reporter expression can be detected within cells that reside central to the malformed myocardium (Fig. 7). The data suggests that maturation of the endocardial layer is arrested when macrophage function is defective, rather than an earlier effect on cardiac precursor cell specification.

### 2.5 Disruption of cardiogenesis by macrophage-morpholino can be rescued by tissue replacement

A conventional rescue of the lurp1-MO morpholino defects by co-injection of *lurp1* RNA proved to be impossible because exogenous *lurp1* itself caused gross developmental malformations, notably to the heart, proctodeum and tail (data not shown). This is not perhaps surprising, since lurp1 is a secreted protein that is likely to mediate macrophage function at discrete targets within the embryo and its widespread production might be expected to be deleterious. We therefore sought an alternative method to prove that the defects caused by the *lurp1* morpholino, and also the *spib* morpholino, originate from within the myeloid domain.

**Fig. 8:**
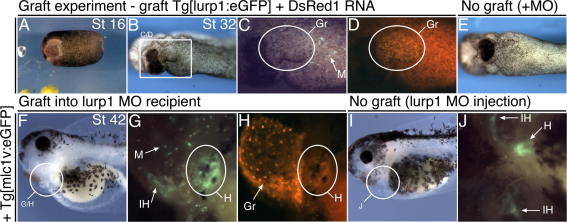
Tissue replacement surgery recovers heart formation defects of macrophage-morpholinos. (A–D) Anterior-ventral tissue replacement surgery was performed at stage 16 between a donor embryo and a recipient embryo (A, Supplementary Movie 1, see Section 4). The donor carries the *Tg[lurp1:egfp]* transgene and had been injected with DsRed1 RNA into 2/2 blastomeres. The recipient embryo carries the *Tg[mlc1v:egfp]* transgene and was injected with the lurp1-MO into dorsal blastomeres, 16 ng dose. Graft recipient tadpole at stage 32 (B). White rectangle (B) shows position of fluorescence images (C-green, migratory macrophages; D-red, graft tissue). (F–H) The same tadpole now at stage 42 has a beating heart. White circle (F) shows position of fluorescence images, ventral view (G-green, donor-macrophages and recipient-heart; H-red, graft tissue). E, I, J: Sibling tadpole injected with lurp1-MO but was not operated on, shown at stage 32 (E) and stage 42 (I and J). The remnant of myocardial tissue does not form a lumen and does not beat (J). Anterior is to left in all views. Gr, graft; M, macrophage; IH, interhyoid facial muscle; H, heart.

We devised a tissue replacement approach, whereby embryos injected with the lurp1-MO received a wild-type myeloid domain grafted in place of the defective one (Suppl. Movie 1, see Section 4). Graft surgery was performed at stage 16, when there is the maximum physical separation between the myeloid mesoderm on the ventral midline and the more laterally located heart progenitors (Fig. 8A). In addition to injection with morpholino, the recipient embryos carried the *Tg[mlc1v:egfp]* cardiac reporter transgene to allow visualization of the forming (ventricular) myocardium (Smith et al., 2005). Embryos providing donor tissue carried the *Tg[lurp1:egfp]* transgene (Smith et al., 2002, 2007) to monitor the efficiency of macrophage transfer to the recipient and had also been injected with DsRed1 RNA to reveal the full extent of the graft.

By surgically replacing the ventral midline tissue, large numbers of migratory transgenic macrophages could be transferred to the recipient embryo (Fig. 8C and G). The grafted region also took on a trough-like shape characteristic of the ventral midline at stage 32, a morphology that is lacking in lurp1-MO injected embryos (Fig. 8B and E). As the graft recipient tadpoles grew, it was evident that the severity of the heart malformations caused by the lurp1-MO was significantly reduced (Fig. 8G, Suppl. Table 4). The recipient embryos formed beating hearts (42%) that underlay the graft region. Importantly, the heart myocardium was heavily derived from the transgenic recipient embryo (as indicated by GFP fluorescence in the ventricular myocardium), demonstrating that the lurp1-MO does not irrevocably prevent myocardial cells from contributing to a functioning heart (Fig. 8G). Ungrafted, lurp1-MO injected sibling embryos never formed a recognizable heart structure at the morpholino dose used, despite some limited myocardial differentiation as evidenced by GFP fluorescence (Fig. 8J). Myeloid tissue replacement was also repeated for embryos injected with the *spib* morpholino with similar results. Beating hearts were observed in graft recipient tadpoles where none formed in ungrafted, morpholino injected siblings (Suppl. Table 4).

Graft recipient tadpoles did subsequently develop edemas that affected later tadpole development. This presumably resulted from failure or inappropriate connection of the heart to the developing vasculature and may have had many causes, not least disruption caused by the grafting procedure. Nevertheless, the grafting experiments clearly demonstrate that introduction of normal macrophages into *lurp1* and *spib* morpholino-injected embryos results in a rescue of heart morphogenesis, confirming that macrophage in the anterior-ventral region of the embryo are important for normal heart formation.

## 3 Discussion

Many “primitive” macrophages reside within the heart-forming region in *Xenopus* embryos during the period when bilateral myocardial cell layers converge to form a linear heart tube. Although the macrophages are a migratory population, at this stage they underlie the myocardium as the initial wedge-shaped trough is formed. Furthermore, histological analysis also suggests that these cells broadly frame the lateral boundary of the myocardium with the adjacent splanchnic mesoderm.

By using morpholino oligonucleotides to inhibit the differentiation of the myeloid mesoderm into macrophages (*spib*) or impair early macrophage function (*lurp1*), we have now shown that the close, transient association of macrophage and cardiac populations is important for normal heart tube development. Morpholinos targeting either gene yielded substantially similar heart phenotypes; both disrupted formation of the trough-shaped myocardium that precedes the heart tube with catastrophic consequences for subsequent cardiac morphogenesis and function.

### 3.1 Spib mediated effects on cardiogenesis

In *Xenopus* embryos, the ets protein spib is the first transcription factor found to be required for production of primitive macrophages. Using *spib* morpholinos, Costa et al. (2008) demonstrated that macrophages fail to migrate from their site of origin during tailbud stages of development. By using embryos of the *Tg[lurp1:egfp]* line to monitor macrophage production in living embryos, we have found that it is possible to sustain the *spib*-mediated block on macrophage production through to tadpole stages, albeit at a reduced frequency. This has enabled us to correlate the extent of macrophage suppression with the effect on heart formation. That macrophage levels eventually recover significantly in the tadpole is not perhaps surprising since there appears to be significant redundancy in the pathways controlling formation of the later, definitive myeloid lineages (Hibbs et al., 2007).

The disruption to heart formation resulting from *spib* or *lurp1* gene knockdown is not the result of an induced developmental delay since no recovery is observed as development proceeds. Rather it appears to be due to disruption in the morphogenetic steps that regulate formation of the myocardial trough, with the result that subsequent steps in heart formation cannot occur. Amphibian embryos can develop relatively far without normal heart formation, but eventually succumb to the edema that results from the absence of circulation. A surprising observation from our studies was that morpholino injection not only compromised cardiac morphogenesis, but also appeared to reduce the extent of myocardial gene expression. Molecular markers of differentiated myocardium can normally be detected from stage 27 (Latinkic et al., 2004; Mohun et al., 2000) and their expression is robust by stage 31, the stage at which injected tadpoles were analysed. No comparable delay in differentiation was evident in other tissues, as judged by comparable external morphology of injected and control tadpoles.

Might this then mean that terminal differentiation of adjacent myeloid and myocardial mesoderm domains are linked in *Xenopus*, such that compromise of one affects the other? It would be interesting to establish if other experimental interventions that have been shown to inhibit cardiac induction or differentiation might act at least in part through an effect on macrophage development. In this respect, it is noteworthy that in addition to the well-established effect on heart formation, morpholinos targeting “cardiac” GATA transcription factors in zebrafish embryos have recently been shown to have parallel effects on the myeloid domain (Peterkin et al., 2009).

### 3.2 Macrophages and cardiac morphogenesis

What role might macrophages play during *Xenopus* heart morphogenesis? One established role for macrophages in embryogenesis is as key producers and modifiers of the extra-cellular matrix (ECM) environment (Olofsson and Page, 2005). The dependency of cardiogenesis on the ECM is well established, with early myocardial migration in zebrafish requiring fibronectin deposition and later organogenesis within cardiac chambers dependent on an ECM “cardiac jelly” (Peal et al., 2009; Trinh and Stainier, 2004). In *Xenopus*, macrophages underlying the myocardium prior to heart tube formation (stage 29, Fig. 4B and E) may therefore be an important source of ECM deposition or modification. Such ECM may be important in facilitating the normal ventral-ward movement of myocardium away from the endoderm that occurs as the myocardial trough forms. *Xenopus* macrophages are known to produce three key matrix metalloproteinases (mmp) that modify the composition of the embryonic ECM (Tomlinson et al., 2008). Morpholino knockdown of these macrophage mmp, notably *mmp18*, has been shown to inhibit macrophage migration during tailbud stages of development and it will be interesting to determine if such macrophage-impaired embryos subsequently develop myocardial morphogenesis defects similar to those reported here.

A second well-described function of macrophages during embryogenesis is the phagocytosis of apoptotic corpses that arise from programmed cell death (PCD) (Henson and Hume, 2006). However, there is no clear evidence that PCD contributes to shaping the myocardial layer itself prior to tube formation. Perhaps the most likely phagocytic macrophages near the developing heart are those found at the boundary between myocardium and more lateral splanchnic mesoderm (Fig. 4B) (Smith et al., 2002), where developmental remodelling will ultimately give rise to the dorsal mesocardium structure that supports the tubular heart.

A third possible function of macrophages in heart formation may be trophic support of vascular endothelial/endocardial growth. The centre of the forming myocardial trough in *Xenopus* is initially filled with macrophages, but rapidly shifts to encompass endocardial precursors adhering to only a few remaining macrophages (stages 29–31, Fig. 4E and F). Cells with an endocardial morphology could not be detected in any of our experiments that perturbed macrophage differentiation and function, although *smad3* reporter expression typical of the endocardial lineage was identified. Perhaps a tentative comparison can be made between this situation and the extensively studied macrophages associated with adult tumors (TAMs) that actively stimulate angiogenic vessel production (Lin and Pollard, 2007). Macrophages might function to stimulate endocardial growth using mechanisms analogous to the catastrophic behaviour of TAMs in malignant cancers. The alternative possibility is that this population of primitive macrophages have some lineage relationship with the subsequent endocardial tissue. We identify *Xenopus* primitive macrophages by hybridization for *mpo* (*pox2*) expression and activity of the *Tg[lurp1:egfp]* reporter line. It would seem most unlikely that cells expressing such markers of mature myeloid function would switch fate to form the final endocardial lining of the heart. Indeed, no residual GFP-fluorescence in the endocardium is ever detected with the transgenic line (data not shown).

### 3.3 Lurp1 mediated effects on cardiogenesis

The precise function of the Ly6 protein encoded by *lurp1* is unknown but our knockdown studies demonstrate that its expression is important for macrophage-mediated function in heart morphogenesis. The Ly6 domain forms a conserved core structure that can tolerate wide sequence variation within the three protruding loops or fingers. It is found not only within Ly6 proteins, but also in a related family of snake venom toxins, the uPAR protein and in the extra-cellular domains of the BMP/activin receptors and their membrane-bound, BAMBI inhibitors (pfam clan CL0117) (Finn et al., 2010). Aside from the SRP pre-sequence and structural residues, there is poor conservation of Ly6 sequences between mammals and lower vertebrates and lurp1 is the sole, secreted Ly6 protein thus far characterized in *Xenopus*. Its closest mammalian homolog is actually a GPI-anchored protein Ly6G6E (Mallya et al., 2006), based on similarity of non-structural amino acids. Thus far, some of the secreted mammalian Ly6 proteins studied have possessed acetylcholine receptor binding activity (Levitin et al., 2008) but nothing is known about the activity of lurp1. As a secreted protein, a first step towards understanding its role might be preparation of a transgenic line that expressed tagged lurp1 protein under control of its endogenous *lurp1* promoter. This would enable study of the distance that lurp1 might act from macrophages and also identify lurp1 target cells.

### 3.4 Is macrophage involvement in cardiac morphogenesis conserved

The presence of macrophages within the heart-forming region has now been reported in many animal species, but their contribution to cardiogenesis has not so far been explored. In *Drosophila* larvae, one major route of plasmatocyte macrophage migration follows the length of the dorsal midline as cardioblasts align there to form the contractile dorsal vessel (Wood et al., 2006; Wood and Jacinto, 2007). The *Drosophila* macrophage deficient *pvr* mutant is lethal but malformations of the heart have as yet, not been defined. In zebrafish embryos, the origin of primitive macrophages lies adjacent to the heart, similar to the arrangement in *Xenopus* (Herbomel et al., 1999). Myocardial morphogenesis defects have been described in zebrafish mutants of endocardial development, although the genetic interventions employed would have had parallel effects on the primitive macrophages (Holtzman et al., 2007).

In the mouse, primitive macrophages are known to be produced in the extra-embryonic yolk sac, some distance from the cardiac crescent mesoderm, and the presumed migration routes they follow to enter the embryo proper have not been determined (Ovchinnikov et al., 2008; Palis et al., 2001). Moreover, the endocardium has a closer cell lineage relationship to the myocardium than it appears to in lower vertebrates, hinting that different mechanisms might drive early heart tube formation (Misfeldt et al., 2009). Nevertheless, the absence of any null mutation that specifically ablates primitive macrophages in the mouse embryo has precluded analysis of their function (Dai et al., 2002; Lichanska et al., 1999; Ovchinnikov, 2008) or embryonic targets. To date, therefore, the possibility of a functional interaction between primitive macrophages and the embryonic heart has not been explored. However, the transitory association of these cells with early cardiac tissue appears to be common to a wide range of species, raising the intriguing possibility that heart formation may be an early and evolutionarily conserved target of macrophage function.

## 4 Experimental procedures

### 4.1 *Xenopus laevis* exon boundary sequences of spib and lurp1 genes

*X. laevis* *spiba* 4 kbp intron 1 was amplified from genomic DNA (Advantage Polymerase, Clontech) using oligonucleotide primers; spib-1910, spib-1912. The exon boundaries were sequenced to enable morpholino design; GenBank:GU451723. No genomic products could be amplified using primers to the *X. laevis* *spibb* allele. The *X. laevis* *lurp1* exon boundary sequences were obtained from two *lurp1* genomic DNA clones, one of which was used previously to isolate the *lurp1* promoter (Smith et al., 2002). The intronic sequences of the clones are subtly different (allele variants a and b); GenBank:GU451724, GenBank:GU451725. Oligonucleotides used for sequencing; L1-1735, L1-1737, L1-1738, L1-1739, L1-1740. The *X. tropicalis* *lurp1* gene resides on JGI 4.1, scaffold_159, 73251-70050 (cDNA Image:7003814).

### 4.2 Morpholino design

**Supplementary Fig. 6:**
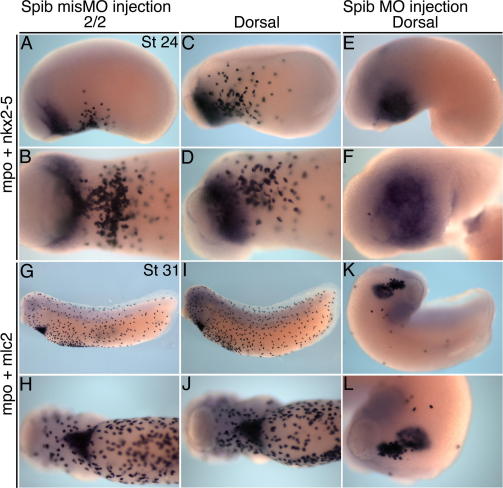
*Spib mismatch morpholino does not affect myeloid cell production.* (A–D) Stage 24 embryos injected with the mismatch control morpholino, Xlspiba-e1i1misMO, into both blastomeres at the two-cell stage (A and B), 40 ng dose, or into dorsal blastomeres (C and D). Embryos hybridized to *mpo* and *nkx2-5*. (E and F) Sibling embryo injected with the active *spib* morpholino, Xlspiba-e1i1MO, into dorsal blastomeres at 40 ng dose. (G–J) Stage 31 tadpoles with the same sequence of mismatch control *spib* morpholino and hybridized to *mpo* and *mlc2*. (K and L) Sibling tadpole injected with the active *spib* morpholino. Myeloid cell production and embryo development is unaffected by the mismatch control morpholino whereas the active morpholino inhibits myeloid development.

**Supplementary Fig. 7:**
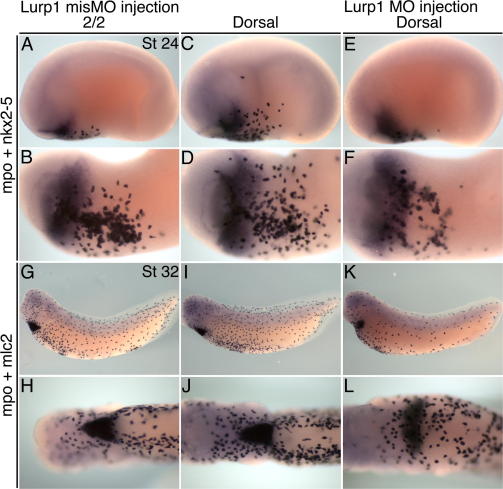
*Lurp1 mismatch morpholino does not affect Xenopus development.* (A–D) Stage 24 embryos injected with the mismatch control morpholino, lurp1-misMO, into both blastomeres at the two-cell stage (A and B), 12 ng dose, or into dorsal blastomeres (C and D). Embryos hybridized to *mpo* and *nkx2-5*. (E and F) Sibling embryo injected with the active *lurp1* morpholino into dorsal blastomeres at 12 ng dose. (G–J) Stage 31 tadpoles with the same sequence of mismatch control *lurp1* morpholino and hybridized to *mpo* and *mlc2*. (K and L) Sibling tadpole injected with the active *lurp1* morpholino. Myeloid cell production and embryo development is unaffected by the mismatch control morpholino whereas the active morpholino causes macrophage migration from lateral positions and defective (broader) heart myocardium morphogenesis.

The sequences of antisense morpholino oligonucleotides (MO) (Gene-Tools) that interfere with gene RNA splicing and also ones that inhibit protein translation are listed in Suppl. Fig. 1. Morpholinos were designed to target the *spib* exon 1-intron 1 boundary, the *lurp1* translation initiation sequence and also the *lurp1* exon 2-intron 2 boundary. Negative control morpholinos containing mismatched sequences are also listed (Suppl. Figs. 1, 6 and 7). A lurp2-MO containing five different mismatches to *lurp1* that was designed to the closest homologous Ly6 gene in *Xenopus* (Image:6872870) causes a distinct early phenotype to formation of the neural plate (data not shown).

### 4.3 Injection of morpholino oligonucleotides into *Xenopus* embryos

Standard procedures were used for the micro-injection of MOs into *Xenopus* embryos (Sive et al., 2000). Morpholino concentrations of 4 or 8 ng/nl were employed, with typically 2 nl injected per embryo blastomere. The following abbreviations are used to denote injection of the different blastomeres; 2/2, both blastomeres injected at the two-cell stage; Dorsal, both dorsal blastomeres at 4-cell stage, or both dorsal-vegetal blastomeres at 8-cell stage; Ventral, both ventral blastomeres at 4-cell stage, or both ventral-vegetal blastomeres at 8-cell stage. The total injection dose is quoted for each experiment; hence 16 ng MO injected refers to 8 ng in each blastomere. The dorsal blastomere injections distribute morpholino to embryo regions that produce both myeloid mesoderm and the heart. The control ventral injections yield morpholino in more posterior-ventral tissue. Injection at the 8-cell stage reduces the concentration of morpholino deposited in neural tissue. This was preferred for experiments with *lurp1*, which has a neural expression domain whose function has not been explored in this study. All morpholinos were additionally injected in embryos into 2/2 blastomeres to confirm consistency of the phenotypes with the dorsal blastomere injections (Suppl. Fig. 2, Fig. 5).

### 4.4 Target specificity of the translation-inhibiting lurp1-MO morpholino

A plasmid construct was made that contained the 5′-UTR and adjacent coding nucleotides of *lurp1* fused in frame with a nuclear localized form of eGFP (pCS2-L1-eGFP-nuc). A control construct featured the 5′-most sequence of the related *lurp2* sequence (pCS2-L2-eGFP-nuc). Capped RNA (200 pg) for the fusion constructs was injected equatorially into two-cell stage embryos. Subsequently, 16 ng of lurp1-MO (plus 4 ng rhodamine-B dextran 10,000, Invitrogen) was injected into half of the embryos at the four-cell stage, into the same embryo region. The fluorescence of all resulting embryos was assessed over time. Oligonucleotides were used to prepare DNA linkers that were cloned using *Age*1-*Nco*1 sites into the 5′-cds of an original pCS2-eGFP-nuc clone; L1-1295 and L1-1296 annealed for the *lurp1* clone, L2-1297 and L2-1298 for the *lurp2* clone.

### 4.5 Tg[smad3:egfp] endocardial cell transgenic reporter line

A 1.5 kbp *X. laevis smad3* gene promoter fragment (GenBank:HQ890547) was isolated from a genomic DNA library (Stratagene) and cloned upstream of eGFP. Transgenic frogs were generated as described previously (Smith et al., 2006). The transgenic line yields strong eGFP expression that peaks in the endocardium around stage 32, plus head, eye, pronephros, somite and notochord domains.

### 4.6 Tissue replacement surgery on morpholino injected embryos

All injection experiments used eggs from a single wildtype female frog. Embryos that were to receive the surgical graft were fertilized with *Tg[mlc1v:egfp]* (or *Tg[mlc2:gfp]*) testes (Smith et al., 2005) and injected with *lurp1* or *spib* morpholinos into dorsal blastomeres (16 ng lurp1-MO, or 32 ng spib-MO doses). Embryos that were to donate tissue graft were fertilized with *Tg[lurp1:egfp]* testes (Smith et al., 2002, 2007) and injected with DsRed1 RNA into 2/2 blastomeres, 800 pg total dose. Tissue replacement surgery was performed between stage-matched, stage 16 embryos using a Gastromaster (Xenotek). Embryos were placed ventral side up, in troughs formed in agarose lined Petri dishes, in 0.75× NAM. A piece of tissue from the anterior ventral midline, absolutely adjacent to the newly forming cement gland, was transferred from the donor embryo to the recipient embryo (Suppl. Movie 1). The tissue graft included ectoderm and mes/endoderm layers. A cover slip fragment was placed over the troughs to hold the transferred graft and the embryos in place. Movies were recorded of every surgical graft to assess their quality. Twelve hours after surgery, embryos were transferred to fresh dishes and 0.1× NAM. Paired donor and recipient embryos were photographed at stages 32, 40 and 42. The normal table of *Xenopus* development was used to stage embryos (Nieuwkoop and Faber, 1956).

### 4.7 Oligonucleotide sequences

L1-1295, 5′-CCGGTAGAAACCCACTATGAAAACTGCAGC.

L1-1296, 5′-CATGGCTGCAGTTTTCATAGTGGGTTTCTA.

L2-1297, 5′-CCGGTATACACCCACTATGACCACTGCTGC.

L2-1298, 5′-CATGGCAGCAGTGGTCATAGTGGGTGTATA.

L1-1469, 5′-AGACGGATCCGGCGCGCCCGCCACCATGAAAACTGCAGTTGTTTTGGTCGT.

L1-1470, 5′-ATGTTCTAGAGCCGTTGCAGAGGTCAGTAGAACAGCACC.

L1-1735, 5′-ATGAAAACTGCAGTTGTTTT.

L1-1737, 5′-GCTTTGAAGTGTCGGAAAAG.

L1-1738, 5′-ACAAAGCACAGAGTGGA.

L1-1739, 5′-ACTCGTGGGTGCATCAC.

L1-1740, 5′-CAGCACCGAACATTAGGATC.

spib-1910, 5′-CTCTCAAAATGCTCAGCCTGGATTCACTC.

spib-1912, 5′-CTGGCAGCTGAAAGAGTTGTCAAAC.

## Appendix A Supplementary data

Supplementary video 1 *Tissue replacement surgery on a morpholino injected embryo.* A piece of anterior-ventral tissue is surgically removed from one stage 16 embryo and added to a second, recipient embryo. The donor and recipient embryos are the actual examples presented in Fig. 8. There is a left-sided bias to the removal of tissue from the recipient, which is reflected in the leftward position of the tissue graft.
