# Supplementary figures and images for: Early cardiac morphogenesis defects caused by loss of embryonic macrophage function in Xenopus
Source: Mech Dev. 2011 May;128(5-6):303–15. doi: 10.1016/j.mod.2011.04.002 (PMC3157588; doi:10.1016/j.mod.2011.04.002)

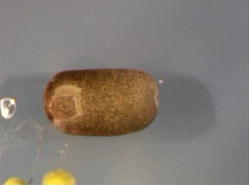

Supplement: Supplementary video 1 — Tissue replacement surgery on a morpholino injected embryo. A piece of anterior-ventral tissue is surgically removed from one stage 16 embryo and added to a second, recipient embryo. The donor and recipient embryos are the actual examples presented in Fig. 8. There is a left-sided bias to the removal of tissue from the recipient, which is reflected in the leftward position of the tissue graft. [file mmc1.jpg]
